# Paraoxonase 1 knockout rats have impaired T cell development at the CD4/CD8 double-negative to double-positive transition stage

**DOI:** 10.1038/s41598-018-32780-w

**Published:** 2018-09-27

**Authors:** Lin Bai, Guiying Shi, Yuanwu Ma, Li Zhang, Feifei Guan, Xu Zhang, Yanfeng Xu, Houzao Chen, Lianfeng Zhang

**Affiliations:** 1 Key Laboratory of Human Disease Comparative Medicine, Ministry of Health, Institute of Laboratory Animal Science, Chinese Academy of Medical Sciences and Comparative Medical Center, Peking Union Medical College, Beijing, China; 2 Department of Biochemistry and Molecular Biology, State Key Laboratory of Medical Molecular Biology, Institute of Basic Medical Sciences, Chinese Academy of Medical Sciences and Peking Union Medical College, Beijing, China

## Abstract

Paraoxonase 1 (PON1) is a high-density lipoprotein-associated enzyme that performs multiple physiological activities. Previous studies suggest that PON1 plays an anti-inflammatory role in the cardiovascular system, although its roles in hematopoiesis and adaptive immunity have not been clarified. To investigate the impact of PON1 on the immune system, we generated PON1-knockout (PON1^−/−^) rats using the CRISPR/Cas9 system. The thymus was smaller in PON1^−/−^ rats than that in wild-type (PON1^+/+^) rats. Furthermore, analysis of thymocyte development revealed diminished total T cell numbers and a decrease in CD4^+^, CD8^+^ and double-positive T cells in peripheral blood and thymus from PON1^−/−^ rats. This may be due to a block in the transition of T cells from the double-negative to the double-positive stage. We also showed that the activation of p38 MAPK phosphorylation contributed to the increased apoptosis and defective T cell development in PON^−/−^ rats. Therefore, our results indicate that PON1 functions as a novel regulator of T cell development.

## Introduction

T cell development is a complex biological process in the thymus that combines differentiation, proliferation, apoptosis and selection. T cell differentiation requires control of the balance of survival and death by extrinsic and intrinsic factors^1^. Cell apoptosis plays a critical role in thymocyte development. Immature thymocytes undergo random rearrangement of their T cell receptor genes and display the successfully rearranged protein products on the cell surface. Some of these cells are then positively selected for further differentiation on the basis of their T cell receptors. The remaining cells, up to 95% of the CD4 and CD8 T cell precursors, die by apoptosis^2,3^.

Paraoxonase-1 (PON1) is a high-density lipoprotein (HDL)-bound enzyme that prevents low-density lipoprotein (LDL) oxidation by macrophages and has been implicated in protection against atherosclerotic lesions. Reduced PON1 activity is associated with disorders such as diabetes, cardiovascular disease, rheumatoid arthritis, cancer and acute infections^4–6^. Multiple *in vitro* studies in animals and human cells have demonstrated the anti-inflammatory and anti-oxidative function of PON1^7–10^. PON1 was shown to decrease monocyte chemotaxis and adhesion to endothelial cells and to inhibit monocyte-to-macrophage differentiation, while PON1 deletion was associated with overexpression of adhesion molecules^11,12^. Furthermore, PON1 activity correlates with CD4^+^ T cell numbers and the immune status of HIV-1-infected individuals^13,14^. These observations suggest an anti-inflammatory role for PON1 *in vivo*, although the mechanism is not fully understood.

To investigate the role of PON1 in the regulation of immune system, we generated PON1-knockout (PON1^−/−^) rats and used this model to demonstrate that PON1-deficiency leads to reduced T cell numbers in the peripheral blood (PB), spleen and thymus. The search for an underlying mechanism revealed a partial block in thymocyte development at the CD4/CD8 double-negative (DN) to double-positive (DP) stage, with the reduction in T cell numbers due to an increase in DN thymocyte apoptosis. Mechanistically, we demonstrated that p38 phosphorylation was upregulated in DN and DP PON1^−/−^ thymocytes. Eliminating p38 phosphorylation in PON1-deficient thymocytes rescued the survival and developmental defects, while activating p38 in WT rats induced a thymocyte defect. Therefore, in this study, we tested the hypothesis that PON1 is required to prevent excessive cell apoptosis by inhibiting the activation of p38 signaling pathway in the development of T cells.

## Materials and Methods

### Animals

Sprague Dawley rats were purchased from Beijing Vital River Laboratories Animal Center and were maintained in a standard facility. PON1^−/−^ rats were generated on a Sprague Dawley background as described previously^15^. In brief, a mixture of mCas9 and single guide RNA (sgRNA) targeting PON1 were injected into fertilized eggs, which were then transferred to pseudopregnant Sprague Dawley rats. The following oligonucleotides were used for sgRNA preparation: A. 5′-TAGGATCGAAACTTTTTATTCC-3′; B. 5′-AAACGGAATAAAAAGTTTCGAT-3′. The paired oligonucleotides were annealed and cloned into the pUC57-sgRNA expression vector (Addgene, #51132). The sgRNA and Cas9 mRNA were prepared using an *in vitro* transcription kit (Am1354 and Am1345, respectively). For the analysis of mutations, genomic DNA was extracted from the tail-snips of 7-day-old rats using the phenol-chloroform method and purified by alcohol precipitation. PON1 mutations were detected by PCR using the primer pair: PON1-1-S: 5′-tgttctgggactgatgattaagtg-3′; PON1-1-A: 5′-tccttctccagtactgtgtctatctg-3′. The mutations were confirmed by Sanger sequencing.

All animal experiments were approved by the Animal Care and Use Committees of the Institute of Laboratory Animal Science of Peking Union Medical College (ILAS-GC-2015-002) and conducted in accordance with the National Institutes of Health Guide for the Care and Use of Laboratory Animals.

### Flow cytometry

Cells were harvested from the thymus, spleen, peripheral blood (PB) and bone marrow (BM) of PON1-knockout (PON1^−/−^) and wild-type (PON1^+/+^) rats. The spleen and thymus were excised immediately, washed with saline, and weighed. Spleens and thymuses were gently homogenized in a glass homogenizer and cells were suspended in sterile PBS. The cells from PB were applied to blood red cell lysis (BD Biosciences). The cells from BM were isolated by flushing both tibias and femurs with sterile PBS. All the cells were isolated by filtration across a sterile nylon mesh and stained for 30 min at 4 °C with the following fluorophore-conjugated antibodies: PE-conjugated anti-CD3 (G4.18), APC-conjugated anti-CD4 (OX35), PE-Cy7-conjugated anti-CD8a (OX8), PerCP-Cy5.5-conjugated anti-CD90.1 (HIS51), PE-conjugated anti-macrophage marker (HIS36), APC-conjugated anti-CD45RA (OX33), PE-conjugated anti-CD25 (OX39) and FITC-conjugated anti-CD44H (OX-49). All antibodies were obtained from eBiosciences and BioLegend Inc. (San Diego, CA, USA). Data were acquired by a FACS Aria II (Becton Dickson) and analyzed using FlowJo software.

### Cell proliferation and cell apoptosis analyses

For cell proliferation analysis, thymus cells were first stained for the indicated cell surface markers. After fixation and permeabilization (BD Biosciences), the cells were stained with FITC-conjugated anti-Ki-67 and 7-AAD (eBiosciences, San Diego, CA). Data were acquired by a FACS Aria II (Becton Dickson) and analyzed using FlowJo software.

For cell apoptosis analysis, thymus cells were first stained for the indicated surface markers. After washing with buffer, the cells were then stained with anti-Annexin V and 7-AAD (eBiosciences). Data were acquired by a FACS Aria II (Becton Dickson) and analyzed using FlowJo software.

### Reactive oxygen species (ROS) production analysis

Thymus cells were incubated with DCFH-DA (Beyotime Company, China) at 37 °C for 20 min. DCFH-DA diffuses passively into the cells, where it is deacetylated by esterases to form non-fluorescent 2′,7′-dichlorofluorescein (DCFH). The amount of fluorescence emitted correlates with the quantity of ROS in the cell. Data were acquired by a FACS Aria II (Becton Dickson) and analyzed using FlowJo software.

#### Transplantation study

In transplantation studies, bone marrow cells (2 × 10^6^) from donor rats (PON1^+/+^, PON1^−/−^ and GFP-rat) were transplanted into lethally irradiated recipient rats. At 2, 4, and 6 weeks after transplantation, the donor-derived chimeric cells in the peripheral blood and thymus was analyzed by flow cytometry. Data were acquired by a FACS Aria II (Becton Dickson) and analyzed using FlowJo software.

### Terminal deoxynucleotidyl-transferase-mediated dUTP nick-end labeling (TUNEL) assays

TUNEL assays were performed using *In Situ* Cell Death Detection kits (Roche) according to the manufacturer’s instructions. In brief, paraffin-embedded sections (5-μm thickness) form PON1^+/+^ and PON1^−/−^ rats thymus were fixed in 10% formalin, deparaffinized, rehydrated and rinsed with PBS for 10 min. The sections were incubated with proteinase K at 37 °C for 30 min, and then incubated with the TUNEL reaction mixture at 37 °C for 1 h. The sections were rinsed three times with PBS and then added to the converter-POD at 37 °C for 30 min. The DAB substrate was added at 25 °C for 10 min. Sections were then mounted under a glass coverslip and analysed under a light microscope.

### Western blot analysis

For the sample, the thymus were excised immediately, washed with saline, and weighed. Thymuses were gently homogenized in a glass homogenizer and cells were suspended in Dulbecco’s modified Eagle’s medium and added the chelerythrine chloride and SB203580. For Western blot analysis, cell lysates were prepared in RIPA buffer (50 mM Tris–HCl, pH 8.0, 100 mM NaCl, 0.1% SDS, 0.5% sodium deoxycholate, 1% NP-40), and a protease inhibitor cocktail (Roche). Total protein concentrations were measured using a BCA kit, and proteins were separated in clarified cell extracts by 12% SDS–polyacrylamide electrophoresis. The proteins were the transferred to a nitrocellulose membrane (Millipore) and incubated at 4 °C overnight with primary antibodies for the detection of PON1 (Abcam, Cambridge, MA), p38 MAP kinase (Cell Signalling), p-p38 MAP kinase (Thr180/Tyr182) (Cell Signalling), and Fas (Santa Cruz, USA). HRP-conjugated anti-mouse and anti-rabbit secondary antibodies were used for detection of immunoreactive bands and visualized using a chemiluminescent detection system (Santa Cruz, USA).

### Immunohistochemical analysis

The thymus was fixed in 4% formaldehyde and mounted in paraffin blocks. The sections were pre-treated using heat-mediated antigen retrieval with sodium citrate buffer for 20 min. Sections were then incubated overnight at 4 °C with primary antibodies for the detection of CD4 (H-370), CD3 (PC3/188 A) and CD8a (H-160) (all from Santa Cruz, USA), and CD45RA (OX-33), CD68 (ED1) and PCNA (PC10) (from Abcam). Immunoreactivity was detected using a HRP-conjugated compact polymer system and DAB was used as the chromogen, Sections were then counterstained with hematoxylin and mounted with DPX.

### Statistical analysis

Data were analysed using Microsoft Excel and GraphPad Prism software. Data were presented as the mean ± standard deviation (SD). Differences between groups of data were analyzed using Student’s *t*-test. *P* < 0.05 was considered to indicate statistical significance.

## Results

### Generation and identification of the PON1 knockout rats using the CRISPR/Cas9 system

**Figure 1:**
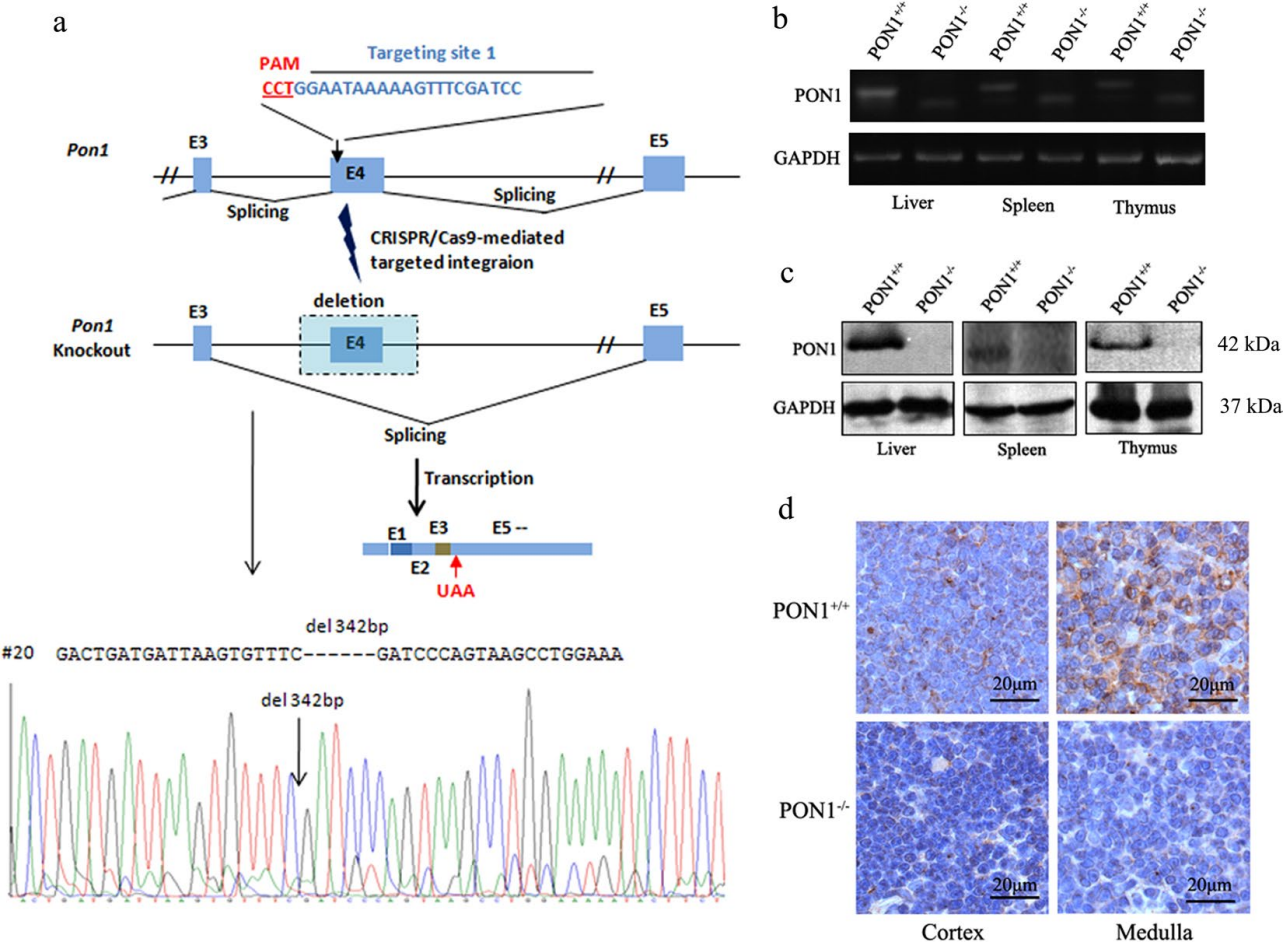
Generation and identification of PON1^−/−^ rats using the CRISPR/Cas9 system. (**a**) Schematic overview of the strategy used to generate PON1^−/−^ rats. (**b**) RT-PCR analysis of PON1 mRNA levels in the liver, spleen and thymus of 2-month-old PON1^−/−^ and PON1^+/+^ rats. (**c**) Western blot analysis of PON1 protein levels in the liver, spleen and thymus of 2-month-old PON1^−/−^ and PON1^+/+^ rats. (**d**) Immunohistochemical analysis of PON1 expression in the cortex and medulla of the thymus in 2-month-old PON1^−/−^ and PON1^+/+^ rats. GAPDH expression was used for normalization. Scale bar = 20 μm.

To investigate the role of PON1 in the immune system *in vivo*, we generated PON1^−/−^ rats using the CRISPR/Cas9 system using the strategy presented in Fig. 1a. Specific sgRNA targeting PON1 exon 4 was transcribed *in vitro* and mixed with Cas9 mRNA; the mixture was then microinjected into Sprague-Dawley rat zygotes. The resulting rats carried a 342-bp deletion in the *PON1* gene. At 2-months-old, the disruption and absence of PON1 in the liver, spleen and the thymus of PON1^−/−^ rats was confirmed by RT-PCR analysis of the RNA (Fig. 1b) and Western blot analysis of the protein (Fig. 1c). Immunohistochemical analysis showed defective expression of PON1 in the cortex and medulla of the thymus from PON1^−/−^ rats compared with that in WT rats (Fig. 1d). These data confirmed stable PON1 deficiency in the PON1^−/−^ rats.

### PON1^−/−^ rats exhibited reduced thymocyte numbers

**Figure 2:**
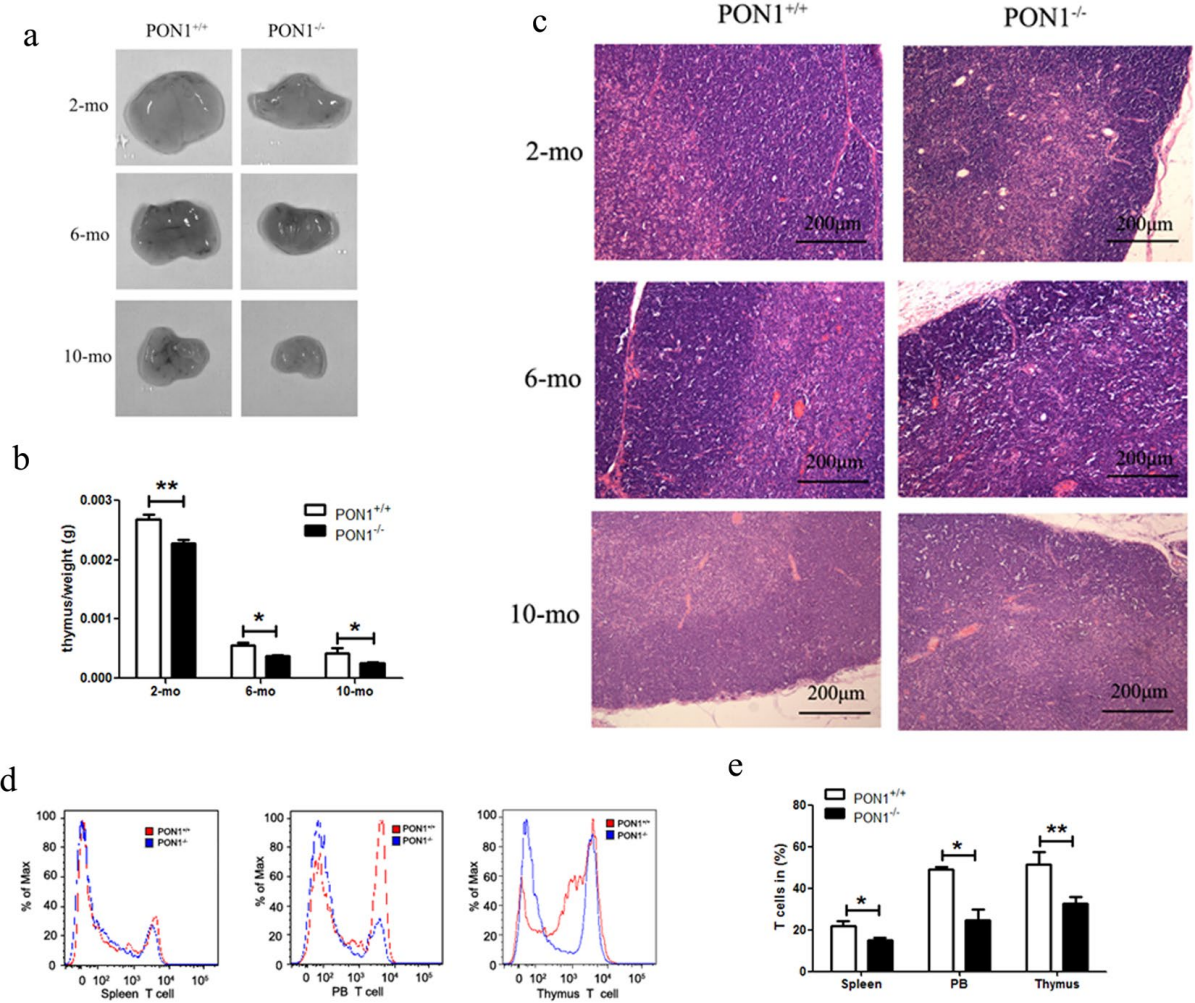
Reduced percentage of thymocytes in PON1^−/−^ rats. (**a**) Images of the thymus from 2, 6 and 10-month-old PON1^−/−^ and PON1^+/+^ rats (*n* = 5 per group). (**b**) Weight of the thymus from PON1^−/−^ and PON1^+/+^ rats aged 2-months (*n* = 9 per group), 6-months (*n* = 6 per group) and 10-months (*n* = 5 per group) old. (**c**) Hematoxylin and eosin staining of thymus from PON1^−/−^ and PON1^+/+^ rats aged 2-months, 6-months and 10-months (*n* = 3 per group). Scale bar = 200 μm. (**d**) Representative histograms of T cells numbers in the spleen, peripheral blood (PB), and thymus from PON1^−/−^ and PON1^+/+^ rats aged 2 months. (**e**) Flow cytometric analysis of the percentage of T cells in the spleen, PB, and the thymus from 2-month-old PON1^−/−^ and PON1^+/+^ rats. Data represent the mean ± SD of three independent experiments (*n* = 5 per group). *P < 0.05; **P < 0.01.

To study the effect of PON1 function on the different hematopoietic elements, we compared the percentages of immune cells from the PB, spleen, BM and thymus in 2-month-old PON1^−/−^ and PON1^+/+^ rats. Flow cytometric analyses indicated that there were no significant differences in the percentage of B cells in the BM and PB of PON1^−/−^ and PON1^+/+^ rats. However, the percentage of B cells was slightly decreased in the spleen of PON1^−/−^ rats compared with that of the corresponding PON1^+/+^ compartment (Fig. S1a,b). The percentages of granulocytes and macrophages were higher in the BM and PB from PON1^−/−^ rats compared with those in the corresponding PON1^+/+^ compartments (Fig. S1c,d). We also found that the percentage of CD68^+^ (macrophage) cells was increased in the thymus from PON1^−/−^ rats compared with that in the PON1^+/+^ rats (Fig. S1e). Based on these differences in the T lymphocyte numbers between PON1^−/−^ and PON1^+/+^ rats, we analyzed the morphology and weight of the thymus in age and sex-matched PON1^−/−^ and PON1^+/+^ rats (*n* = 10). We found that the thymus was smaller in PON1^−/−^ rats compared with that in the PON1^+/+^ rats (Fig. 2a). Furthermore, the weight of PON1^−/−^ rats was approximately 50% lower than that of the PON1^+/+^ rats at the age of 10 months (Fig. 2b). To further confirm these results, we investigated changes in the pathology of the thymus from PON1^−/−^ and PON1^+/+^ rats (*n* = *3*) at the ages of 2, 6, and 10 months. Compared with the PON1^+/+^ littermates, the thymus from PON1^−/−^ rats were small, with a thin cortex (Fig. S2a,c). Vacuoles caused by phagocytosis of apoptotic cells by macrophages were observed in the 10-month-old PON1^−/−^ rats (Fig. 2c), while there were no apparent differences in the pathological changes observed in the PON1^−/−^ and PON1^+/+^ rats (Fig. S2b). Flow cytometric analysis revealed a significant reduction in the percentage of T cells in the spleen, PB and thymus of PON1^−/−^ rats compared with that in the PON1^+/+^ rats (Fig. 2d,e). Thus, our results indicated a reduction in the number of thymocytes in PON1^−/−^ rats.

### PON1^−/−^ rats exhibit a block in T lymphocyte maturation at the DN stage

**Figure 3:**
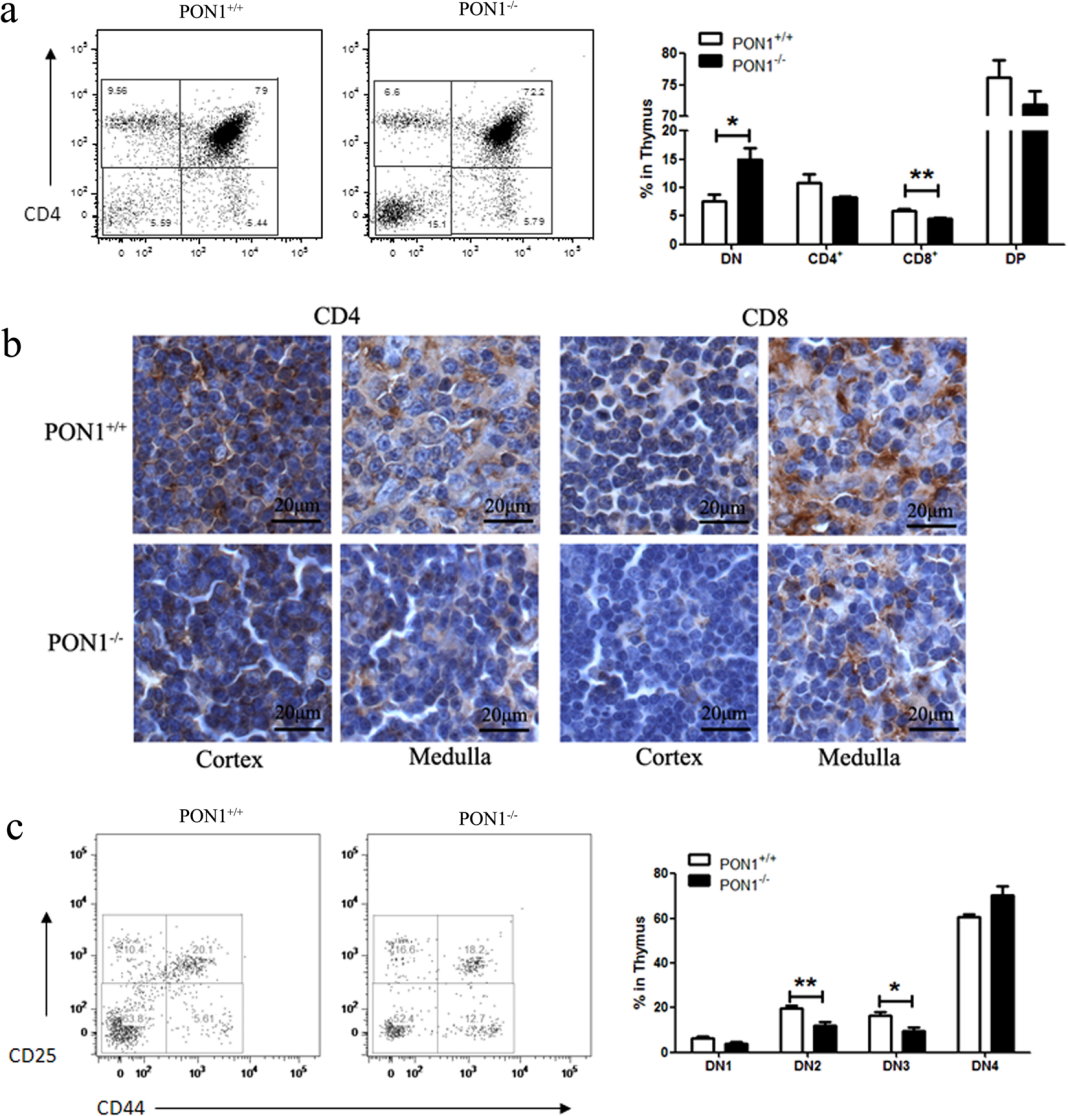
PON1-deficiency blocks T lymphocyte maturation at the DN stage. (**a**) Representative staining profiles for T cells and the percentages of double-negative (DN), CD4^+^, CD8^+^ and double-positive (DP) in the thymus of 2-month old PON1^−/−^ and PON1^+/+^ rats. (**b**) Immunohistochemical analysis of CD4^+^, CD8^+^ cells in the cortex and medulla of the thymus of 2-month-old PON1^−/−^ and PON1^+/+^ rats. Scale bar = 100 μm. (**c**) Representative staining profiles for DN cells and the percentage of DN1, DN2, DN3 and DN4 cells in the thymus from 2-month-old PON1^−/−^ and PON1^+/+^ rats. Data represent the mean ± SD of three independent experiments. *P < 0.05; **P < 0.01.

To study the function of PON1 in T cell development, we analyzed the thymocytes harvested from PON1^−/−^ and PON1^+/+^ rats by flow cytometry. Compared with the PON1^+/+^, the percentages of CD8 and CD4 single-positive (SP) and double-positive (DP) T cells were reduced in PON1^−/−^ rats, while the percentage of DN cells was increased (Fig. 3a). These data indicated defective T lymphocyte development in PON1^−/−^ rats, with the cells arrested in the DN stage. These findings were confirmed by immunohistochemical analysis showing reduced numbers of CD4^+^ and CD8^+^ cells in the cortex and medulla of the thymus of PON1^−/−^ rats compared with that of PON1^+/+^ rats (Fig. 3b).

According to the expression of the markers CD44 and CD25, the DN stage of T cell development can be divided into the following four stages: DN1 (CD44^+^CD25^−^), DN2 (CD44^+^CD25^+^), DN3 (CD44^−^CD25^+^) and DN4 (CD44^−^CD25^−^). Flow cytometric analysis showed that the percentages of DN1, DN2 and DN3 cells were decreased in PON1^−/−^ rats compared with those in PON1^+/+^ rats, while the percentage of DN4 cells was increased (Fig. 3c). These results indicated that PON1 may influence the transition from the DN4 stage in the development of T cells. Thus, PON1-deficiency results in disturbed T cell development characterized by massively reduced thymic cellularity and a partial DN4 block.

### PON1 influences T lymphocyte apoptosis

**Figure 4:**
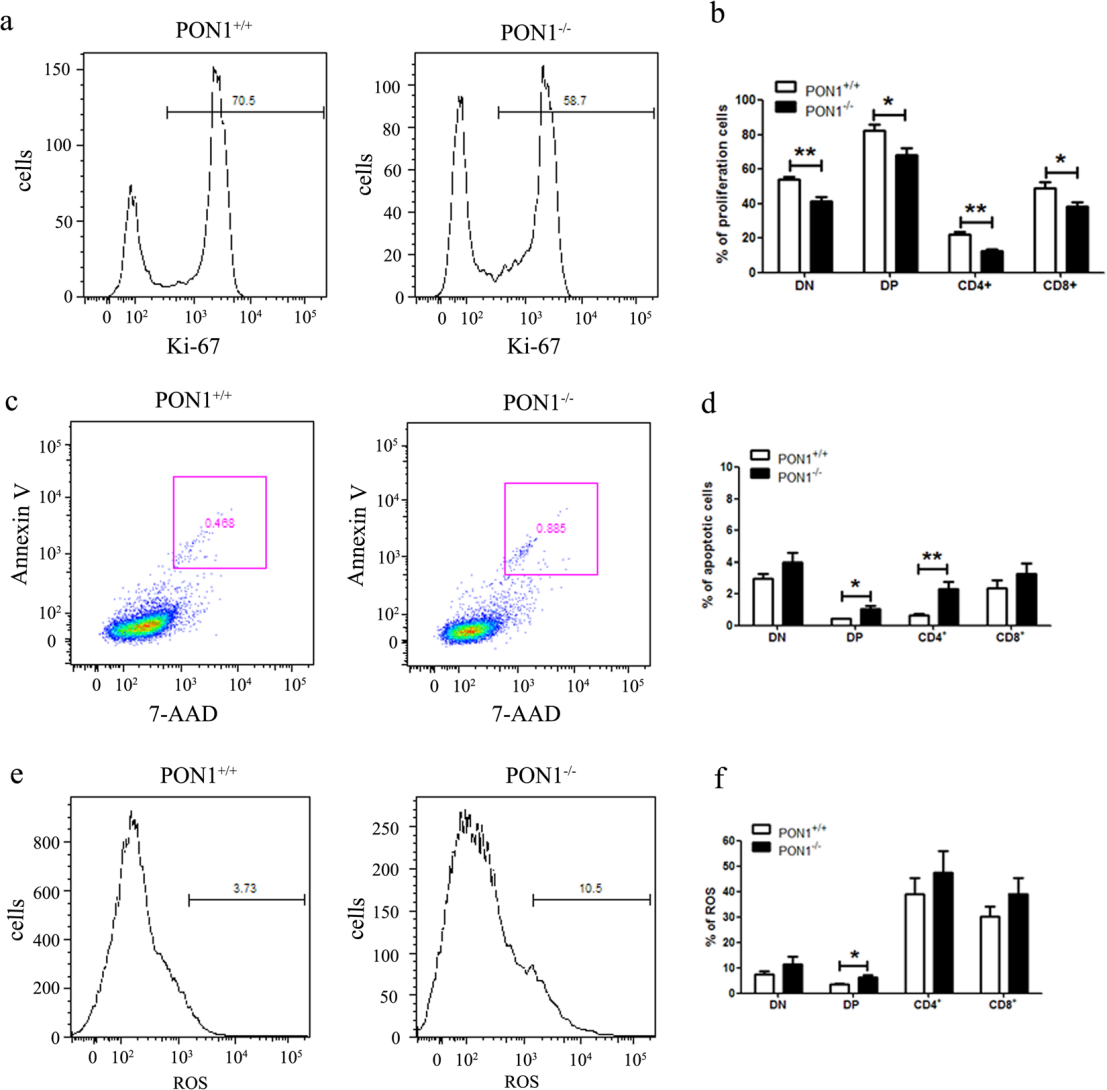
PON1 influences T lymphocyte proliferation and apoptosis. (**a**) Representative profiles for Ki-67 staining of T cells in the thymus of 2-month-old PON1^−/−^ and PON1^+/+^ rats. (**b**) The percentage of proliferating cells among the DN, CD4^+^, CD8^+^ and DP populations from the thymus. (**c**) Representative profiles for 7-AAD and Ki-67 staining of T cells in the thymus of 2-month-old PON1^−/−^ and PON1^+/+^ rats. (**d**) The percentage of apoptotic cells among the DN, CD4^+^, CD8^+^ and DP populations from the thymus. (**e**) Representative ROS profiles of T cells in the thymus from 2-month-old PON1^−/−^ and PON1^+/+^ rats. (**f**) The percentage of ROS-positive cells among the DN, CD4^+^, CD8^+^ and DP populations from the thymus. Data represent the mean ± SD (*n* = 5 per group). *P < 0.05; **P < 0.01.

To determine the mechanisms underlying the role of PON1 in defective T cell development, we performed flow cytometric analysis of the proliferation and survival of T cells. Identification of Ki-67 as a marker of proliferating cells (Fig. 4a) showed a lower percentage of proliferating cells in the thymus of PON1^−/−^ rats compared with that in PON1^+/+^ rats (Fig. 4b). Based on our observation of vacuoles caused by phagocytosis of apoptotic cells by macrophages in the 10-month-old PON1^−/−^ rats, we analyzed T cell apoptosis by flow cytometric analysis of annexin V and 7-AAD staining (Fig. 4d). The data showed that the percentage of apoptotic cells was increased in the CD4 SP and DP cells (Fig. 4d).

Oxidative stress is a critical determinant of hematopoietic stem cell lifespan and self-renewal. The loss of HSC quiescence frequently correlates with increased levels of cellular ROS. We analyzed the levels of ROS in thymocytes from 2-month-old PON1^−/−^ and PON1^+/+^ rats by flow cytometry (Fig. 4e). Compared with PON1^+/+^, the levels of ROS were higher in the DP cells of PON1^−/−^ rats, while there were no significant differences in the levels of ROS in the SP and DN cells (Fig. 4f). These results indicated that PON1 is required to protect thymocytes against apoptosis.

### Reduction in T cell numbers in PON1^−/−^ rats does not depend on the thymic microenvironment

**Figure 5:**
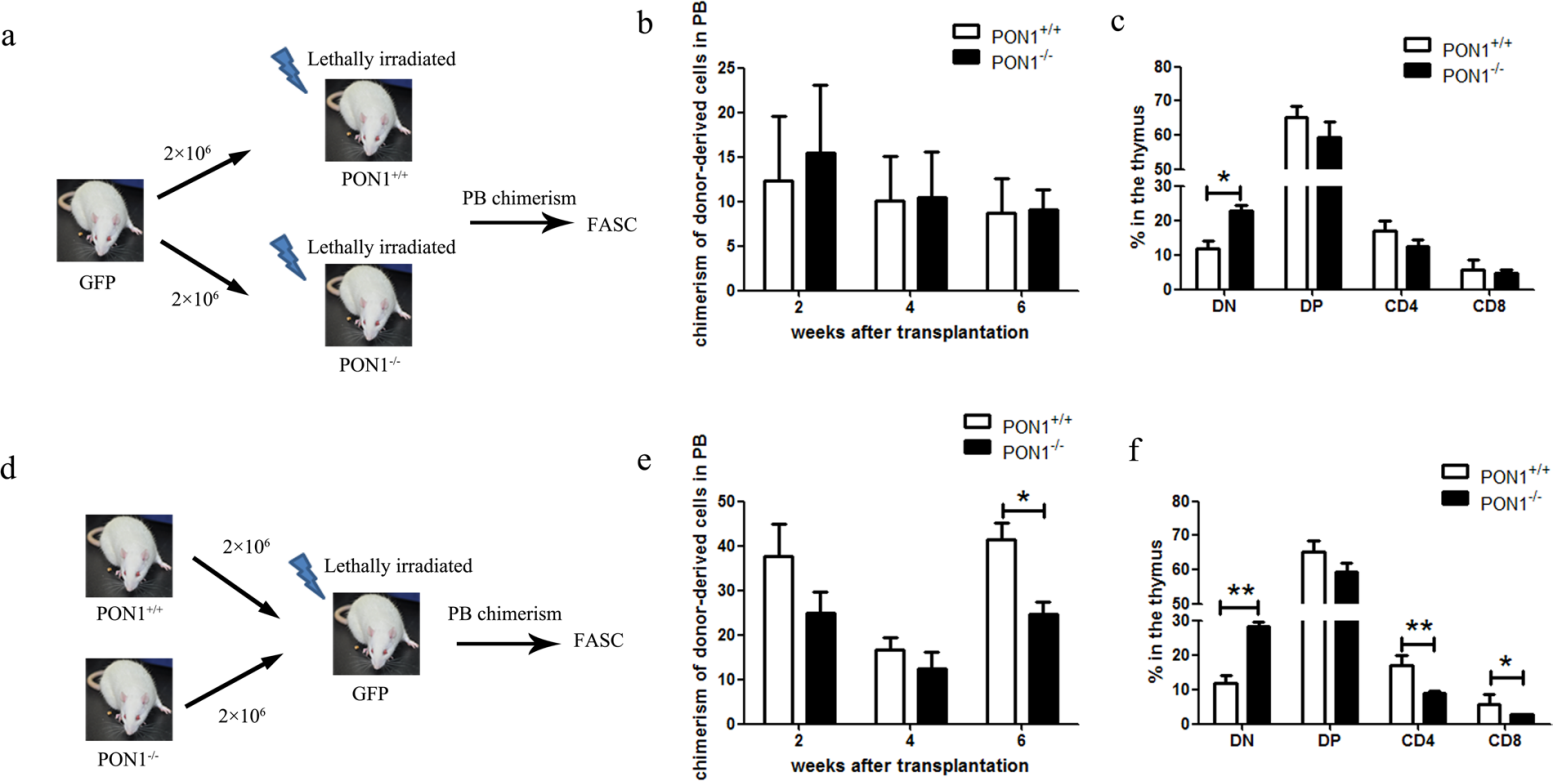
PON1^−/−^ results in a reduction in T cell numbers that is not dependent on the thymic microenvironment. (**a**) Schematic overview of the strategy for bone marrow transplantation. (**b**) Chimerisms of donor-derived cells in the peripheral blood (PB) of irradiated recipient rats at 2, 4, and 6 weeks after transplantation with either PON1^+/+^ or PON1^−/−^ BM cells (*n* = 9). (**c**) The percentage of GFP-derived T cells in the PB of PON1^+/+^ or PON1^−/−^ recipient rats 6-weeks after transplantation (*n* = 9). (**d**) Schematic overview of the strategy for bone marrow transplantation. (**e**) Chimerisms of donor-derived cells in the PB of irradiated recipient rats from 2, 4, and 6 weeks after transplantation with GFP BM cells (*n* = 5). (**f**) The percentage of PON1^+/+^- or PON1^−/–^derived T cells in the PB of GFP recipient rats 6 weeks after transplantation (*n* = 5). Data represent the mean ± SD. *P < 0.05; **P < 0.01.

T cell development in the thymus is a highly regulated process and the survival of T lymphocytes is dependent on both extrinsic and intrinsic signals. To investigate the effect of PON1 on the thymic microenvironment, we transplanted BM cells from two-month-old GFP transgenic rats into lethally irradiated PON1^−/−^ or PON1^+/+^ rats (Fig. 5a). At 2, 4 and 6 weeks after transplantation, GFP^+^ cell chimerism was detected in the PB, with no significant difference between the PON1^−/−^ or PON1^+/+^ transplanted rats (Fig. 5b). At 6 weeks after transplantation, analysis of the percentages of T cells showed decreased percentages of SP and DP cells in PON1^−/−^ transplanted rats compared with the percentages of the corresponding populations in PON1^+/+^ transplanted rats, while the percentage of DN cells was increased (Fig. 5c); however, these changes were not statistically significant. We also transplanted BM cells from 2-month-old PON1^−/−^ or PON1^+/+^ rats into lethally irradiated GFP transgenic rats (Fig. 5d). At 2, 4 and 6 weeks after transplantation, GFP^+^ cell chimerism was detected in the PB, with lower levels of chimerism in the PON1^−/−^ transplanted rats that were more obvious at 6 weeks post-transplantation (Fig. 5e). Analysis of the T cells at 6 weeks after transplantation showed decreased percentages of CD4 and CD8 SP cells in PON1^−/−^ transplanted rats compared with those in PON1^+/+^ transplanted rats, while the percentage of DN cells was increased (Fig. 5f). These data suggested that the changes in T cells associated with PON1 deletion are not dependent on changes in the thymic microenvironment.

### PON1^−/−^ accelerates apoptosis via the p38 MAPK signaling pathway

**Figure 6:**
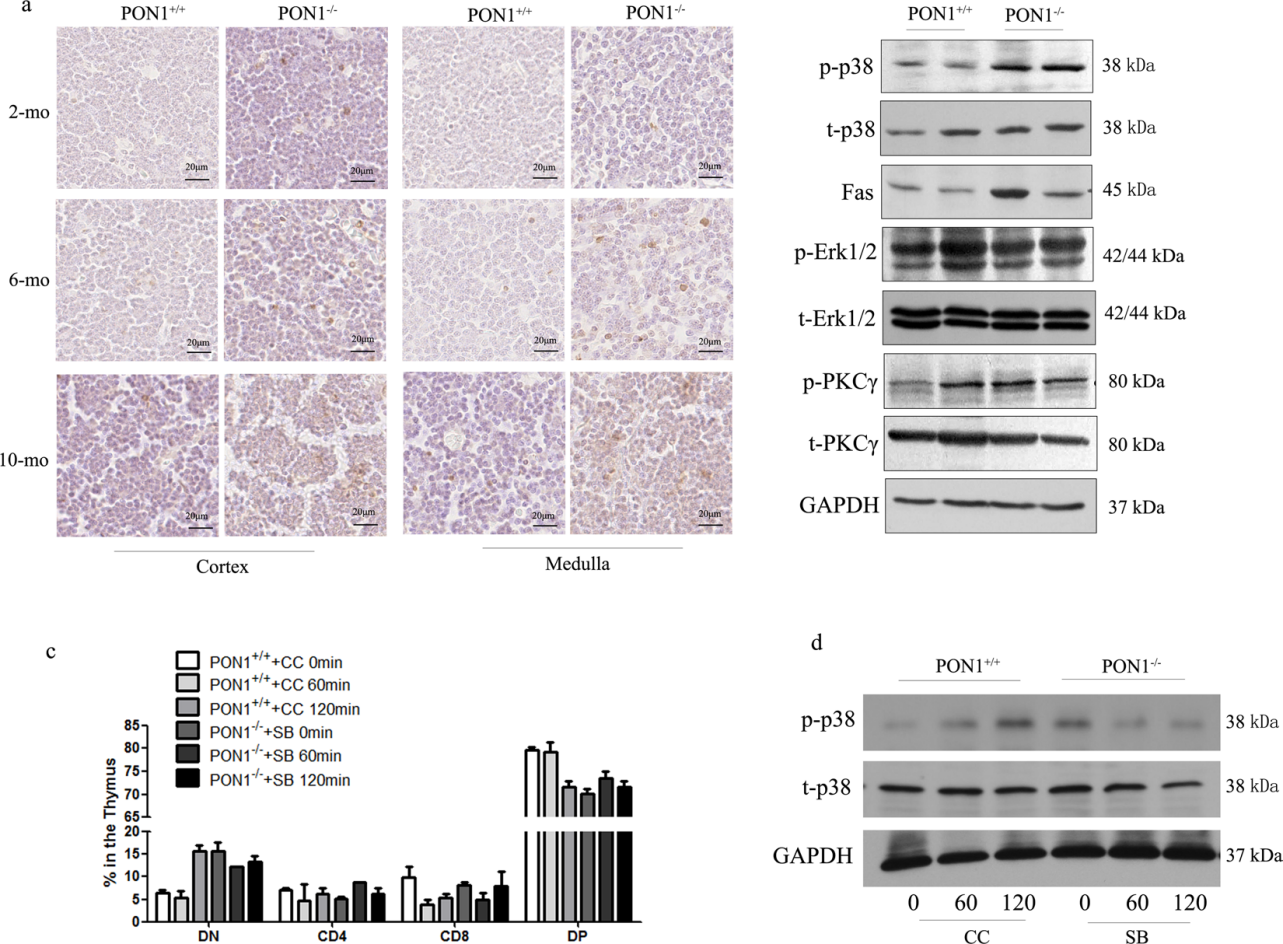
PON1^−/−^ accelerates thymocyte apoptosis via the p38 MAPK signaling pathway. (**a**) TUNEL assays of thymus sections from PON1^+/+^ and PON1^−/−^ rats aged 2, 6 and 10 months. Scale bar = 20 μm. (**b**) Western blot analysis of apoptosis-associated genes in the thymus from PON1+/+ and PON1^−/−^ rats aged 2 months. (**c**) Flow cytometric analysis of the percentage of indicated cells in the thymus from PON1^+/+^ and PON1^−/−^ rats aged 2 months. (**d**) Western blot analysis the expression of p38 MAPK. CC (chelerythrine chloride): p38 MAPK activator and SB (SB203580): p38 MAPK inhibitor. (*n* = 3 rats per group). The experiment was repeated three times.

Several mechanisms could contribute to the defects in thymocytes detected in PON1-KO rats. To confirm that the loss of T cells in the PON1^−/−^ rats was due to apoptosis, we performed TUNEL analysis of cell apoptosis at the single cell level. The results showed that the frequency of apoptotic cells increased in the PON1^−/−^ rats at the ages of 2, 4 and 6 months (Fig. 6a). To investigate the signaling mechanism, we analyzed the expression of p38, Erk1/2, and PKCγ in the PON1^+/+^ and PON1^−/−^ rats. There were no significant differences in the expression of Erk1/2, and PKCγ between the PON1^+/+^ and PON1^−/−^ rats, while increased expression of p38 phosphorylation and the downstream molecule Fas was detected in the PON1^−/−^ thymocytes (Fig. 6b).

To further confirm the signaling pathway involved in the effects of PON1-deficiency, we used the p38 MAPK activator chelerythrine chloride and the inhibitor SB203580 to stimulate the thymocytes from PON1^+/+^ and PON1^−/−^ rats. Flow cytometric and Western blot analyses indicated that p38 activation in PON1^+/+^ rats decreased the percentages of CD4, CD8 and DP T cells, and inhibiting p38 phosphorylation in PON1^−/−^ rats reduced the survival and developmental defects (Fig. 6c,d). These findings indicated that PON1 prevents excessive cell apoptosis by inhibiting activation of the p38 signaling pathway.

## Discussion

PON1 performs anti-inflammatory functions, including inhibiting the production and secretion of pro-inflammatory cytokines in macrophages^11^, suppressing IFN-γ production from CD4^+^ T cells^14^. Furthermore, the activity of PON1 relates to the number of CD4^+^ T cells in HIV-1-infected individuals^13^. However, the mechanism underlying the anti-inflammatory function of PON1 is not fully understood. In our study, we generated PON1^−/−^ rats and found a smaller thymus and decreased thymocyte numbers compared with the PON1^+/+^ rats. The search for an underlying mechanism revealed a partial block in thymocyte development at the DN-to-DP point. In addition, T cell numbers were reduced due to an increase in DN thymocyte apoptosis. The apoptosis of thymocytes in the thymus is regulated by different MAPKs, including Erk1/2, JNK and p38. Compared with PON1^+/+^ rats, we demonstrated that the phosphorylation of p38 was upregulated in DN and DP PON1^−/−^ thymocytes, although there was no significant difference in the levels of Erk1/2. *In vitro*, elimination of p38 phosphorylation in PON1^−/−^ thymocytes rescued the survival and developmental defects, while these defects were induced by p38 activation in PON1^+/+^. Therefore, we conclude that apoptosis is induced in PON1^−/−^ thymocytes by activation of the p38 signaling pathway.

PON1 is synthesized and expressed predominantly in the liver from where it is transferred and localized in multiple tissues via HDLs^4^. Western blot and immunohistochemical analyses confirmed PON1 protein expression in the thymus and PON1 expression at the mRNA level was detected by RT-PCR. These findings indicated that PON1 is synthesized and expressed in the thymus although at much lower levels than those in the liver.

T cell differentiation requires control of the balance of cell production and death by a combination of extrinsic and intrinsic factors. To investigate the effect of PON1 on the thymic microenvironment, we transplanted BM cells from two-month-old GFP transgenic rats into lethally irradiated PON1^−/−^ and PON1^+/+^ rats. The results showed that there was no significant difference in GFP^+^ cell chimerism between PON1^−/−^ and PON1^+/+^ transplanted rats. In contrast, when BM cells from 2-month-old PON1^−/−^ or PON1^+/+^ rats were transplanted into lethally irradiated GFP transgenic rats, GFP^+^ cell chimerism was decreased in the PON1^−/−^ transplanted rats, an effect that was more obvious at 6-weeks post-transplantation. These data suggest that deletion of PON1 does not influence the thymic microenvironment.

Apoptosis plays an important role in T cell development^16^. A large number of immature thymocytes are generated in the thymus, some of which differentiate, while others die by apoptosis due to either negative selection, or failure to receive any selection signals^17^. Flow cytometric and TUNEL analyses showed increased numbers of apoptotic thymocytes in the PON1^−/−^ rats at 2, 4 and 6 months. In terms of the signaling pathway, p38 phosphorylation as well as expression of the downstream molecule Fas were increased in the PON1^−/−^ thymocytes compared with PON1^+/+^ thymocytes.

Thus, PON1-deficiency resulted in blocked thymocyte development at the DN-to-DP stage, with a reduction in the number of T cells due to an increase in DN thymocyte apoptosis. Mechanistically, p38 phosphorylation was upregulated in DN and DP PON1^−/−^ thymocytes. Thus, our study indicates that PON1 is required to prevent excessive cell apoptosis by inhibiting activation of the p38 signaling pathway.

## Electronic supplementary material

Supplementary figure
