# Supplementary material for: Paraoxonase 1 knockout rats have impaired T cell development at the CD4/CD8 double-negative to double-positive transition stage
Source: Sci Rep. 2018 Sep 27;8:14457. doi: 10.1038/s41598-018-32780-w (PMC6160460; doi:10.1038/s41598-018-32780-w)
Supplement: Supplementary file 1 — Supplementary figure [file 41598_2018_32780_MOESM1_ESM.pdf]

Paraoxonase 1 knockout rats have impaired T cell development at the CD4/CD8 double-negative to double-positive transition stage

Lin bai[1], Guiying Shi[1], Yuanwu Ma[1], Li Zhang[1], Feifei Guan[1], Xu Zhang[1], Yanfeng Xu[1], Houzao Chen[2] and Lianfeng Zhang[1]*

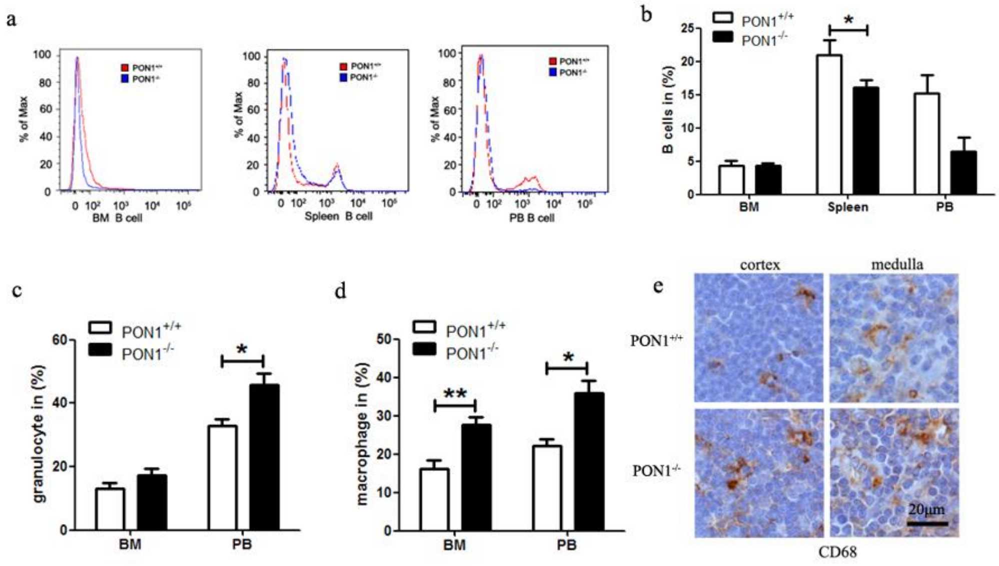

Figure S1. a Representative histograms of B cells in the bone marrow (BM), spleen, and peripheral blood (PB) from PON1-/- and PON1+/+ rats aged 2 months. **b** Flow cytometric analysis of the percentage of B cells in the BM, spleen, and PB of PON1-/- and PON1+/+ rats aged 2 months. **c, d** Flow cytometric analysis of the percentages of granulocytes and macrophages in the BM and peripheral PB from PON1-/- and PON1+/+ rats aged 2 months. **e** Immunohistochemical analysis of CD68+ cells in the cortex and medulla of the thymus from PON1-/- and PON1+/+ rats aged 2 months. Scale bar = 20μm. Data represent the mean ± SD of three independent experiments (n = 5 per group). *P < 0.05; **P < 0.01.

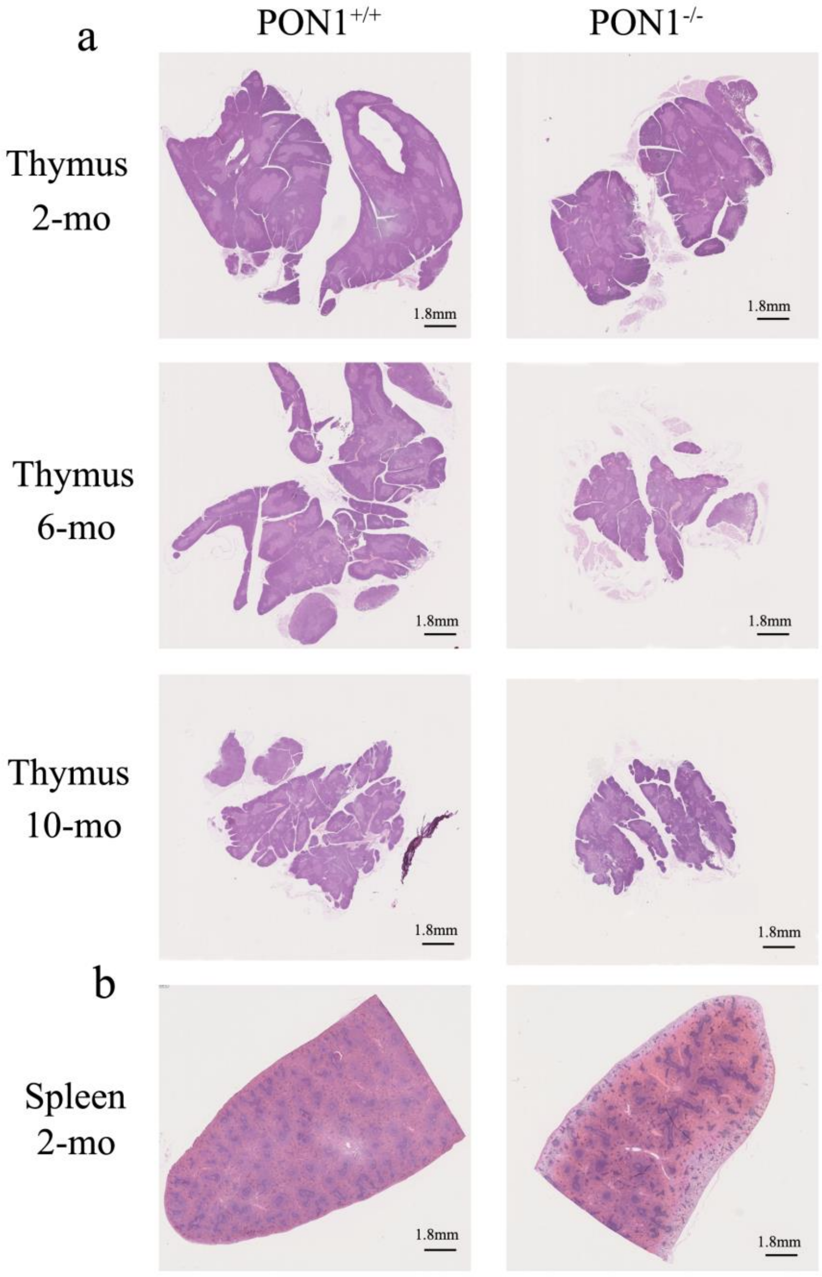

Figure S2. a Histopathological analyses of whole-mounts of thymus sections from PON1+/+ and PON1-/- rats aged 2, 6 and 10 months. **b** Histopathological analyses of whole-mounts of spleen sections from PON1+/+ and PON1-/- rats aged 2 months. Scale bar = 1.8 mm.
